# N-Back Task Training Helps to Improve Post-error Performance

**DOI:** 10.3389/fpsyg.2020.00370

**Published:** 2020-03-11

**Authors:** Qing Li, Quanshan Long, Na Hu, Yancheng Tang, Antao Chen

**Affiliations:** Key Laboratory of Cognition and Personality of Ministry of Education, Faculty of Psychology, Southwest University, Chongqing, China

**Keywords:** working memory training, transfer, post-error performance, post-error slowing, n-back task

## Abstract

Improved performance on working memory (WM) through training has been widely expected to transfer to other domains. Recent studies have proposed that WM training could enhance the autonomous coordination of WM processes. Based on the shared processes between WM and error processing, our present study explored the transfer effect of 15 days of training on post-error performance, during the n-back task, compared to a simple visual search task. Participants were randomly assigned to either the training (*N* = 22) or control (*N* = 18) group. We found that WM training successfully improved WM performance. After training, compared with the control group, the training group showed a significant reduction in post-error slowing (PES); however, post-error accuracy and the flanker effect were not modulated by WM training. Moreover, we observed a significant, negative correlation between the changes in PES and WM from pretest to posttest and classified two groups based on these changes in PES with 70% accuracy. Thus, in our present sample, WM training improved post-error performance. We propose that the skill of controlling information flow, developed during WM training, is transferable to other tasks and discuss the implications of current findings for understanding the generation of PES.

## Introduction

By temporarily storing and manipulating information, working memory (WM) is critical to numerous aspects of cognition (Baddeley, 1986). Improved performance on WM through training has been widely expected to transfer to other performances; however, transfer of WM training to different but related tasks is often either absent or negligible, despite gains in trained WM tasks being consistently large (Au et al., 2014; Melby-Lervåg et al., 2016; Sala et al., 2018). Since WM training has little benefit on everyday cognitive functions that depend on WM, training has been thought to be incapable of increasing the fundamental capacity of WM (Martin and Fernberger, 1929; Chase and Ericsson, 1981; Gathercole et al., 2019). Notably, the processes involved in the transfer tasks adopted by previous studies are quite different, which may be critical to determine the transfer effect. Additional studies have proposed that WM training enhances WM processes, such as updating and inhibition, and that when trained and untrained tasks share these processes, transfer across two tasks may occur (Dahlin et al., 2008; Minear et al., 2016; Soveri et al., 2017). Moreover, Chein and Morrison (2010) found that complex WM training benefits could be generalized to performance on the Stroop task, a common cognitive control task (Stroop, 1935), which enhances both rehearsal in the WM task and the selection of the ink color in Stroop by increasing the shared process of proactive (executive). Actually, the identical elements (IEs) model can provide more specific predictions about the conditions for transfer, which holds that when the elements of the test problem match exactly with those of a practice problem, complete transfer is predicted; otherwise, there is no prediction of transfer (Thorndike, 1922; Rickard and Bourne, 1996). Training typically goes through three stages: cognitive, associative, and autonomous (Fitts and Posner, 1967). Recently, Gathercole et al. (2019) suggested that successful performance during a task relies on the coordination of multiple processes that are isolated when individuals are unfamiliar with the task. Sufficient training may lead to the autonomous coordination of these processes and once established, performance on similar tasks may improve, indicating a transfer effect.

A widely used paradigm in WM training studies is the n-back task, where participants are required to recall a sequence of items and determine whether the current item matches the item presented “n” positions prior (Gevins and Cutillo, 1993). The n-back task involves at least two distinct tasks: processing the information from the current trial while remembering and manipulating the information from prior trials. Importantly, central resources are limited when participants perform the task. To optimally complete the n-back task, participants may split their central resources into two parts, one part to maintain and manipulate the prior trials, while the remainder processes the current trial. During the initial stages of training, the splitting skill may not be adequately established, leading to a worse performance; with training, this skill may gradually become automated, and performance could be improved. According to Gathercole et al. (2019), when participants receiving n-back task training complete alternative tasks (i.e., where the splitting skill can be applied), transfer from WM training to the new task occurs.

Individuals often slow their response after committing an error compared to after an accurate performance, which involves a series of adjustments and is vital for our survival and adaptation (Rabbitt, 1966). Recently, this post-error slowing (PES) had been increasingly studied (Purcell and Kiani, 2016; Ullsperger and Danielmeier, 2016; Buzzell et al., 2017). In previous studies, it was commonly observed that PES differed across participants (Steinborn et al., 2012). PES was found to be modulated by participant alertness and transcranial direct current stimulation intervention on the right dorsolateral prefrontal cortex (Mansouri et al., 2016; Wang et al., 2016). Thus, it is possible to improve post-error performance by experimental manipulation; however, whether WM training can improve post-error performance remains understudied. When PES occurs, participants typically engage in a task similar to the n-back task: they are required to use one part of their central resources to manipulate the error-induced processing from the prior trial while using the remainder to complete the current trial. Therefore, based on the IE model and the view by Gathercole et al. (2019), we predicted that training improvements in the n-back task could transfer to the performance after error.

The present study as an exploratory experiment intended to investigate whether the gains from WM training transferred to task performance following error and the underlying behavioral mechanisms. Participants were randomly assigned into training or control groups. The training group received a 15-day training session with the dual n-back task, while the control group completed simple visual search task training that only utilized low-level perceptual processing, not the splitting skill (Jaeggi et al., 2008; Anderson et al., 2011). To measure post-error performance, we adopted the four-choice flanker task modified by Maier et al. (2008), which was more difficult than typical flanker tasks (Eriksen and Eriksen, 1974), thus ensuring that post-error trials were adequate for analysis. Measures of post-error performance, including post-error reaction time (RT) performance and post-error accuracy performance, were measured before and after training, which allowed us to quantify transfer from n-back task training to post-error performance (Danielmeier and Ullsperger, 2011). The flanker effect was also compared before and after training to determine any transfer to the flanker task. Since the flanker task is used to measure inhibitory control, if specific processes were enhanced during the training, the flanker effect would decrease. According to the IE model and the splitting skill by Gathercole et al. (2019), after training, improved post-error performance should be observed in the training group compared to the control group, but there should be no difference in the flanker effect between the two groups.

## Materials and Methods

### Participants

Forty-two university students from Southwest University in China participated in the present study. All participants were right-handed, had normal or corrected-to-normal vision, and no history of neurological disorders. They were randomly assigned into either the training group (*N* = 22, 4 males, 18 females, mean age = 20.09 years, *SD* = 1.11 years) or the control group (*N* = 20, 10 males, 10 females, mean age = 20.50 years, *SD* = 1.15 years). In addition, both groups did not differ in age [*t*(40) = −1.18, *p* = 0.247], but were significantly different in sex [*t*(40) = 2.24, *p* = 0.032]. Each participant provided written informed consent before the experiment. The study was approved by Southwest University Human Ethics Committee for the Human Research.

### Procedure

**FIGURE 1:**
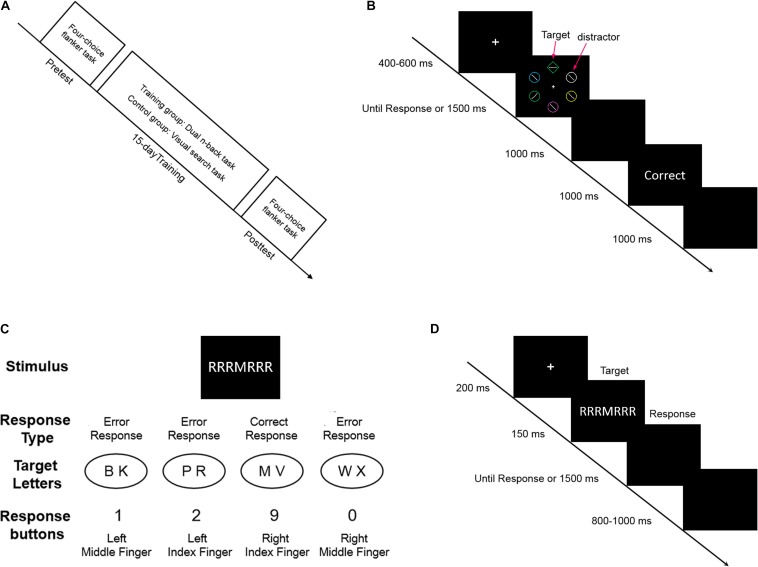
**(A)** Procedure. The tasks and time course of the whole experimental procedure. **(B)** The visual search task. The sequence of events and time course for one trial in the task. **(C)** Stimulus-response mapping in the four-choice flanker task. Each of the four response fingers corresponded to two target letters. In the shown example, if a response was given with the right index finger, it would be classified as a correct response. If a response was given with the remaining fingers, it would be classified as an error response. **(D)** The four-choice flanker task. The sequence of one typical trial in the task.

Participants were seated in a comfortable chair in a quiet testing room. The experiment was conducted using E-Prime software (Psychology Software Tools, Inc., Pittsburgh, PA, United States) and run on a 17-inch Dell monitor (with a refresh rate of 85 Hz and a resolution of 1,024 × 768). Participants sat at a distance of approximately 60 cm from the screen, and the stimuli were presented at the center of the screen. Before training, all participants attended a pretest session on the four-choice flanker task, following which, both groups received a 15-day training session. During the sessions, the training group completed the dual n-back task, while the control group performed the simple visual search task. Fifteen days after the first test, both groups participated in the posttest session on the four-choice flanker task (Figure 1A). To maintain motivation, both groups obtained a similar instruction before each session indicating that participants were required to remember the reaction rules and respond as quickly and accurately as possible (Benedetti et al., 2003; Long et al., 2019).

### Tasks

#### Dual n-Back Task

To train WM, we adopted the same material as described by Jaeggi et al. (2008), but the task parameters and thresholds were adopted from the Default Mode developed by Brain Workshop^1^. Each trial started with a white fixation point in the center of the computer screen, and the eight locations around the white fixation point would randomly and continuously present a visual stimulus (blue square) at a rate of 3 s. The stimulus was presented for 500 ms with an interstimulus interval of 2,500 ms. Simultaneously with the presentation of the visual stimulus, an auditory stimulus of one of eight consonants (c, g, h, k, p, q, t, and w) spoken in a female voice was presented through the headphones and selected on the basis of its distinctiveness. Participants were instructed to discern whether the current stimulus matched the target stimulus presented n trials before. There were two modes of stimulus presentation; one was where the auditory and visual targets appeared in only one modality, and the other was where the two targets appeared in both modalities simultaneously. Their positions were determined randomly. Participants had to simultaneously pay attention to both modalities, and responses were required independently for each. They were required to press the letter “A” on the standard keyboard with the left index finger for a visual target and the letter “L” with the right index finger for an auditory target. For non-targets, participants were not required to respond.

The difficulty level of the task increased with *n*, and the value of *n* was contingent on the individuals’ performance in the previous block. After each block, the participants’ performance was analyzed. If the accuracy of three consecutive blocks was greater than 80%, the level of n for the next block increased by one. It decreased by one if the accuracy was lower than 50%; otherwise, n remained constant. Participants started training with a two-back task with each block consisting of 20 + n trials. A training session was comprised of 20 blocks, which lasted approximately 25 min each day.

#### Visual Search Task

Each trial started with the presentation of the fixation point for a randomly varying interval of 400, 500, or 600 ms, followed by the appearance of the visual stimulus for 1,500 ms (Figure 1B). Participants were instructed to search for the unique rhombus as quickly and accurately as possible, and to identify the target by pressing “F” with the left index finger and “J” with the right index finger for the horizontally and vertically orientated target within the rhombus, respectively. After a blank screen lasting 1,000 ms, feedback was given based on the actual response, and the next trial began after a response–stimulus interval of 1,000 ms. The experiment consisted of six blocks of 36 trials (216 trials in total), which lasted approximately 20 min each day.

#### Four-Choice Flanker Task

The stimulus was composed of eight letters (B, K, P, R, M, V, W, and X) and six neutral symbols (§, $,%, &, #, and ?). In total, 48 incongruent stimuli and 48 neutral stimuli were constructed using all letters and neutral symbols. Participants were instructed to respond to the central target letter and ignore the flankers on each side, and to press “1” with the left middle finger, “2” with the left index finger, “9” with the right index finger, and “0” with the right middle finger (Figure 1C). Each trial began with the appearance of the fixation point for 200 ms, followed by the presentation of the stimulus for 150 ms (Figure 1D). Participants had to respond to the target letter as quickly and accurately as possible within 1,500 ms. When a response was given, the next trial began after a randomly varying response–stimulus interval of 800, 900, or 1,000 ms. The experiment comprised eight blocks consisting of 96 trials each (768 trials in total).

### Statistical Analysis

Statistical analyses were performed using the SPSS software (version 21.0; IBM, Armonk, NY, United States). For all statistical tests, the alpha level was set to 0.05 and the effect sizes referred to partial eta-square values. Outliers, defined as being more than three standard deviations (*SD*) from the individual mean, were excluded in the present study.

#### Training Performance Changes

For the training group, we evaluated the training performance of each participant and each training session and calculated the average level of n-back per training day. We used paired samples *t*-tests to assess individual performance differences between the first and the last training session. For the control group, the average accuracy of all participants per training day was calculated.

#### Post-error Performance in the Four-Choice Flanker Task

Post-error slowing was the difference between the RT of correct trials following error responses (EC) and the RT of correct trials following correct responses (CC; RT_EC_ - RT_CC_). Post-error accuracy was calculated by the accuracy following errors minus the accuracy following correct response (Rabbitt, 1966; Wang et al., 2015a, b). To analyze the effects of training on post-error performance, we used a repeated measures analysis of variance (ANOVA), with Previous Accuracy (correct, error) and Time (pretest, posttest) as within-subject factors and Group (training group, control group) as a between-subject factor.

#### Flanker Effect

To analyze overall performance on the four-choice flanker task, we used an ANOVA, with Time (pretest, posttest) as a within-subject factor and Group (training, control) as a between-subject factor. Further, to analyze the effects of the training on the flanker effect, we used an ANOVA, with Congruency (incongruent, neutral) and Time (pretest, posttest) as within-subject factors and Group (training, control) as a between-subject factor.

#### Correlation Analysis

To investigate the relationship between post-error performance and WM, after controlling for Sex and Age, we conducted a partial correlation analysis with relative changes before and after training in the training group. A stepwise regression analysis was performed with the changes in post-error performance as the dependent variable and the changes in WM, Sex, and Age as predictive factors.

#### Support Vector Machine Classification

According to different behavioral changes between the two groups, we classified the groups (training, control) based on post-error performance. The changes in post-error performance were used as features to differentiate the two groups, and the 1,000 times permutation test was calculated to verify the reliability of the classifications.

## Results

### Participants

Two participants in the control group did not complete the study because they did not have enough time and were therefore excluded. The training (*N* = 22, 4 males, 18 females, mean age = 20.09 years, *SD* = 1.11 years) and control (*N* = 18, 9 males, 9 females, mean age = 20.50 years, *SD* = 1.10 years) groups did not differ in age [*t*(38) = −1.17, *p* = 0.251], but were significantly different in sex [*t*(38) = 2.16, *p* = 0.039).

### Training Performance Changes

**FIGURE 2:**
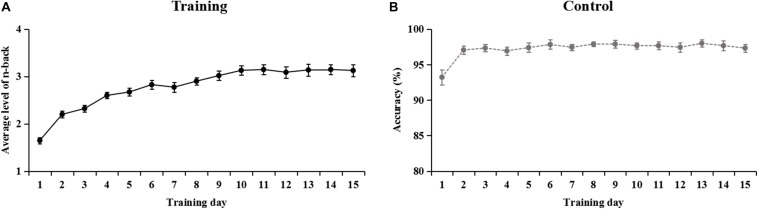
Training performance on the dual n-back task **(A)** and the visual search task **(B)** across training days. For the training group, the average level of n-back is reported for each training session. For the control group, the graph depicts the accuracy per day in the visual search task. Error bars denote standard error.

The training curves for the two groups are shown in Figure 2. For the training group, their performance improved with training time. WM performance significantly increased from the first to the last training day [*t*(21) = −12.42, *p* < 0.001] (Figure 2A). WM training elicited an overall average increase of 1.48 (*SD* = 0.56) (Table 1). For the control group, the average accuracy of participants per training day was all greater than 93% in the visual search task (Figure 2B).

**TABLE 1:** Descriptive statistics.

**Measure**	**Training group**				**Control group**			
	**Pretest**		**Posttest**		**Pretest**		**Posttest**	
	**Mean**	***SD***	**Mean**	***SD***	**Mean**	***SD***	**Mean**	***SD***
Training performance	1.65	0.31	3.13	0.59	0.93	0.05	0.97	0.02
RT on CC trials	670.13	109.90	596.47	138.15	680.13	102.35	599.28	118.96
RT on EC trials	720.86	110.14	603.60	120.45	721.70	110.87	646.87	143.29
Post-correct accuracy	0.93	0.06	0.95	0.04	0.93	0.05	0.93	0.06
Post-error accuracy	0.92	0.05	0.94	0.06	0.89	0.09	0.91	0.08
RT in the flanker task	678.16	110.40	601.30	139.53	689.31	104.99	605.21	119.87
Accuracy in the flanker task	0.93	0.06	0.95	0.04	0.92	0.06	0.92	0.07
RT on incongruent trials	675.17	118.04	600.30	138.33	687.15	107.17	605.28	118.08
RT on neutral trials	660.26	109.17	587.95	139.74	673.74	100.61	585.83	123.96
Accuracy on incongruent trials	0.94	0.06	0.95	0.04	0.92	0.06	0.93	0.06
Accuracy on neutral trials	0.93	0.05	0.94	0.04	0.92	0.06	0.92	0.07

*RT, reaction time; CC, correct responses; EC, error responses; *SD*, standard deviation.*

### Effects of WM Training on Post-error Performance

**FIGURE 3:**
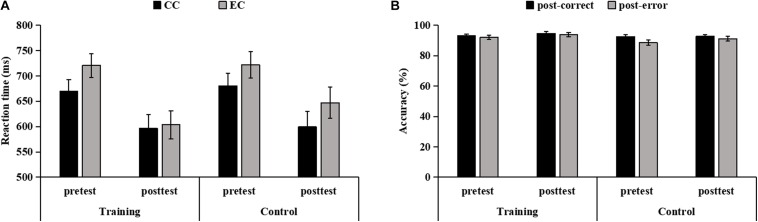
The reaction time (RT) **(A)** and accuracy **(B)** on trials following correct responses and errors for both groups at both pretest and posttest. Error bars denote standard error.

The Previous Accuracy × Time × Group ANOVA of the RT showed main effects of Previous Accuracy [*F*(1,38) = 33.33, *p* < 0.001, η_p_^2^ = 0.47] and Time [*F*(1,38) = 22.48, *p* < 0.001, η_p_^2^ = 0.37], indicating that the RT on EC trials was significantly slower than on CC trials, and that the RT was significantly faster at posttest than at pretest. The interaction between Previous Accuracy × Time was marginally significant [*F*(1,38) = 3.57, *p* = 0.067, η_p_^2^ = 0.09]. *Post hoc* tests revealed that the RT on EC and CC trials were significantly faster at posttest than at pretest (*p* < 0.001). Moreover, the Previous Accuracy × Time × Group interaction was significant [*F*(1,38) = 6.22, *p* = 0.017, η_p_^2^ = 0.14]. *Post hoc* tests revealed that for the training group, the RT on EC trials was significantly slower than on CC trials at pretest [*F*(1,38) = 25.32, *p* < 0.001, η_p_^2^ = 0.40]; however, the RT did not differ between EC trials and CC trials at posttest [*F*(1,38) = 0.38, *p* = 0.541], indicating that the PES disappeared after WM training. For the control group, the RT on EC trials was significantly slower than on CC trials at pretest [*F*(1,38) = 13.91, *p* = 0.001, η_p_^2^ = 0.27) and at posttest [*F*(1,38) = 13.90, *p* = 0.001, η_p_^2^ = 0.27). There was no significant other main effect (*p* = 0.664) or interactions (*ps* > 0.227) (Figure 3A).

The Previous Accuracy × Time × Group ANOVA of the accuracy revealed the main effects of Previous Accuracy [*F*(1,38) = 11.10, *p* = 0.002, η_p_^2^ = 0.23] and Time [*F*(1,38) = 5.06, *p* = 0.030, η_p_^2^ = 0.12), indicating that the accuracy on trials following errors was significantly lower than on trials following correct responses, and that the accuracy was significantly higher at posttest than at pretest. There was no significant other main effect (*p* = 0.191) or interactions (*ps* > 0.116), indicating that there were no differences in accuracy on trials following errors and correct responses from pretest to posttest between the two groups (Figure 3B).

### Effects of WM Training on the Flanker Effect

The Time × Group ANOVA of the RT in the four-choice flanker task showed a main effect of Time [*F*(1,38) = 20.99, *p* < 0.001, η_p_^2^ = 0.36], suggesting that the mean RT of both groups was smaller at posttest than at pretest. The main effect of Group (*p* = 0.825) and the interaction (*p* = 0.838) were not significant. Yet, the mean accuracy did not differ between pretest and posttest (*ps* > 0.099).

**FIGURE 4:**
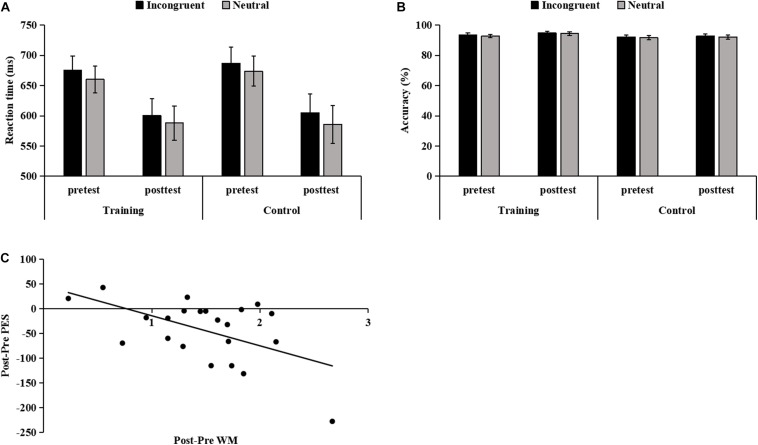
The RT **(A)** and accuracy **(B)** on incongruent and neutral trials for both groups at pretest and posttest. Error bars denote standard error. **(C)** The partial correlations between pretest and posttest changes in post-error slowing (PES) and changes in working memory (WM). The correlation between them is negative because PES reports the pretest to posttest reduction in RT on correct trials following errors compared to following correct responses, while WM denotes the increase in n-back level from pretest to posttest.

The Congruency × Time × Group ANOVA of the RT showed the main effects of Congruency [*F*(1,38) = 23.00, *p* < 0.001, η_p_^2^ = 0.38] and Time [*F*(1,38) = 20.50, *p* < 0.001, η_p_^2^ = 0.35], indicating that the RT on incongruent trials was significantly slower than on neutral trials, and that the RT was significantly faster at posttest than at pretest. There was no significant other main effect (*p* = 0.836) or interactions (*ps* > 0.493). Thus, there was no difference in flanker effect from pretest to posttest between the two groups (Figure 4A).

The Congruency × Time × Group ANOVA of the accuracy showed the main effects of Congruency [*F*(1,38) = 11.09, *p* = 0.002, η_p_^2^ = 0.23] and Time [*F*(1,38) = 4.31, *p* = 0.045, η_p_^2^ = 0.10], indicating that the accuracy on incongruent trials was significantly higher than on neutral trials, and that the accuracy was significantly higher at posttest than at pretest. There were no significant other main effects (*p* = 0.299) or interactions (*ps* > 0.388), indicating that there was no difference in accuracy for incongruent and neutral trials from pretest to posttest between the two groups (Figure 4B).

### Correlation Analysis Results

After controlling for Sex and Age, we found a significant negative correlation between pretest and posttest changes in PES and WM (*r* = −0.553, *p* = 0.011) (Figure 4C). Similarly, the results of the stepwise regression analysis showed that the changes in WM were the only variable that could enter the regression model, which accounted for 25.5% variance of the changes in PES [*F*(1,21) = 8.17, *p* = 0.01].

### Classification Results

The predictive variable was the pretest to posttest change in PES, and the outcome variable was a dichotomous variable (i.e., training and control groups). The trained model resulted in 70% accuracy in classifying the two groups (permutation test: *p* < 0.01).

## Discussion

With an active control group as a strict contrast, the present study investigated whether the improvements elicited by WM training transferred to post-error performance. The training group, who underwent 15 days of n-back task training, demonstrated significantly improved performance in the same n-back task, indicating a significant benefit of WM training. In the flanker task, the PES of the training group was significant at pretest, but not at posttest, while the PES of the control group was significant at pretest and posttest. Thus, PES was eliminated in the training group, but was not affected in the control group. Alternatively, the post-error accuracy was not significant at pretest or posttest for both groups. Therefore, the WM training invoked a comparable RT after error to that after correction at posttest; this improvement was not at the detriment to the decreased accuracy of post-error trials at posttest. We observed a significant transfer effect from WM training to post-error performances, indexed by improved post-error adjustment. Further, the gains from WM training were significantly correlated with decreased PES from pretest to posttest, which could explain 25.5% of the variance in the changes of PES. Moreover, the discrimination model built by machine learning had an acceptable predictive effect on the training and control groups, which resulted in 70% accuracy; however, the flanker effect was not modulated by WM training. Based on the work by Green et al. (2014), we could offer the interpretations for the results. That is, the results were consistent with the theoretical predictions, which supported the IE model and the splitting skill by Gathercole et al. (2019).

If WM training enhances the fundamental capacity of WM, significant transfer to tasks that also depend on WM would occur; however, even from the n-back task to complex span task, the magnitude of transfer was very small (Soveri et al., 2017). In the present study, the mean RT of the neutral condition in the flanker task decreased from pretest to posttest, and the decrease in magnitude in the n-back task was smaller than in the visual search task. The attentional demands are typically low in the neutral condition, where the participants’ performance may primarily rely on their fundamental cognitive function. Thus, if the WM training enhanced fundamental cognitive functions, improvements should be smaller in the training group than in the control group. In the present study, the transfer to post-error performance in the flanker task could not be attributed to enhanced fundamental cognitive functions.

In the influential model by Baddeley and Hitch (1994), manipulation (e.g., executive control) and maintenance are considered two general processes in WM. Chen et al. (2008) proposed that the n-back task may include three more subprocesses: matching, replacement, and shift. Therefore, WM and cognitive control share multiple processes, which leads to the expectation that training WM should facilitate performance in cognitive control tasks (Engle, 2010). Chein and Morrison (2010) found that the training benefits on a complex WM span task could be generalized to performance on a Stroop interference task; however, our results demonstrated that the training gains on the n-back task did not impact the flanker effect. The different results may reflect the divergence between the n-back and complex span WM paradigms (Blacker et al., 2017). Alternatively, we suggested that these basic processes involved in WM tasks may have been sufficiently developed for healthy adults, as they are frequently used in everyday processes (Gathercole et al., 2019). Thus, it is difficult to improve the basic processes of WM exclusively through simple cognitive training, thereby offering understanding as to why the n-back training did not improve the flanker effect.

According to the production system models that represent skilled behavior as sets of production rules incorporating specific knowledge, the complex new activities would be accomplished by combining these rules (Anderson, 1982). So relative to improving fundamental capacity or basic processes, it is more likely that new strategies or skills will be formed during cognitive training. While strategies employed in WM training often differ across participants and do not result in transfer, researchers have recently focused on the new skills acquired in WM training (Minear et al., 2016). Gathercole et al. (2019) suggested that training WM tasks would lead new skills in coordinating distinct processes; moreover, they would gradually become autonomous with training. Some newly formed skills control the flow of information and are independent of task content; however, when they are consistent with those controlling the flow of information in an untrained task, transfer from WM training may occur (Chein and Morrison, 2010; Taatgen, 2013).

In the n-back task, there are two flows of information: one from previous trials, which needs to be maintained and manipulated in WM; and one from current trials, which is current input and needs to be processed immediately. Thus, the skill that divides central resources into two parts to process flow of information should be used in n-back task. With training, the skill would gradually become automated. Importantly, in post-error flanker task, participants encounter similar information flow: the error signal from the previous trial and input information from the current trial. Moreover, the general and specific processes in the n-back task are related to the information from prior trials rather than from current trials; thus, the central resources assigned to the previous information should be larger than those of the current information (Chen et al., 2008). This is similar to the post-error flanker task, where error-related processing from the previous trial occupies more central resources, while the current flanker task is simple and consumes fewer central resources (Jentzsch and Dudschig, 2009; Buzzell et al., 2017; Li et al., unpublished). Thus, the skill of splitting central resources to efficiently utilize differing information flow acquired in the n-back task is applicable in the post-error flanker task and facilitates post-error performance.

Although the current findings highlight the benefits of WM training on PES, there are two limitations that need to be considered. First, we only utilized an active control group, but lacked a real control group where the flanker task was administered before and after a 15-day phase of no training. Therefore, a blank control group should be added in future studies to help better interpret the results. Second, the sample size is limited, which may affect the statistical power in the present study. Therefore, a larger sample size is necessary to ensure adequate statistical power in further studies. Third, individual differences in WM capacity are an important problem that needs to be considered, but it lacked the pre-training measurements between groups in the present study. Accordingly, the future study should collect the data of the WM performance in the control group before training to further support the absence of pre-training differences in WM capacity between groups.

The newly acquired capability of controlling flow of information during n-back task training may help to interpret the transfer to post-error performance (Gathercole et al., 2019). Flow of information is task general (Taatgen, 2013). Although the n-back and post-error flanker tasks differ on task features and processes, the WM processing and error-related processing in both tasks require participants to divide central resources into two parts and assign more central resources to process the information from previous trials and less central resources to process currently available information. Thus, the skills controlling the flow of information developed in n-back task training can be applied to the error-related processing in the flanker task. By understanding the control of information flow can assist in understanding why transfer from WM training may occur. In addition, the present results also suggest that the central bottleneck stage induced by error monitoring in PES can be diminished by effectively splitting central resources (Jentzsch and Dudschig, 2009). This finding further suggests that PES is not only due to strained central resources but also to the interaction between the two components of the central resources for error-related processing and the current flanker task. When participants could use separate central resources to process the two tasks, as shown in the present study, the slowing induced by error can be eliminated, which offers a deeper understanding of the generation of PES; however, empirical data for this skill is insufficient and requires further study to address this phenomenon.

## Data Availability Statement

The datasets generated for this study are available on request to the corresponding author.

## Ethics Statement

The studies involving human participants were reviewed and approved by the Southwest University Human Ethics Committee for the Human Research. The patients/participants provided their written informed consent to participate in this study.

## Author Contributions

QLi designed the tasks. QLi, QLo, and NH collected the data. QLi and YT analyzed the data. QLi and AC wrote the manuscript. All authors approved the final version of the manuscript for submission.

## Conflict of Interest

The authors declare that the research was conducted in the absence of any commercial or financial relationships that could be construed as a potential conflict of interest.

## Footnotes

1 http://brainworkshop.sourceforge.net/
